# A natural whitening alternative from upcycled food waste (acid whey) and underutilized grains (millet)

**DOI:** 10.1038/s41598-023-32204-4

**Published:** 2023-04-20

**Authors:** Mercy Nani, Kiruba Krishnaswamy

**Affiliations:** 1 Division of Food, Nutrition and Exercise Science, University of Missouri, Columbia, MO USA; 2 Department of Biomedical, Biological, and Chemical Engineering, University of Missouri, Columbia, MO 65211 USA

**Keywords:** Sustainability, Nutrition, Chemical engineering

## Abstract

The dairy industry faces a daunting challenge in managing acid whey (AW), a byproduct of Greek yogurt manufacturing that is costly to dispose of and challenging to incorporate into other food products. However, recent studies have demonstrated that AW can be transformed into a viable white powder by encapsulating it in millet flour. Recently, concerns over the safety of the commonly used food-grade whitener titanium dioxide (TiO_2_) have arisen, and the search for an alternative food-whitening agent has become essential. This study evaluated the color attribute, proximate composition, sugar profile, amino acid profile, total phenolic content, antioxidant activity, and antinutrient content of the novel acid whey millet (AWM) powder. The L* values of the AWM powders were significantly higher than TiO_2_ and the rest of the millet formulations. The crude protein content in the AWM powders was significantly (*p* < 0.05) lower when compared to the crude protein content in millet flours. AWM powders had higher lactose levels and retained all major amino acids after spray drying. Macrominerals (P, K, Ca, and Na) and microminerals (Zn and Cu) significantly increased in the AWM powder, while tannin content was reduced in AWM powders. These findings suggest that AWM powder is a white powder that contains a wide range of components with high nutritional value that could be readily incorporated into various applications. In summary, this study provides a valuable contribution to the dairy industry by highlighting the potential of AWM powders as a natural alternative food whitening agent to TiO_2_.

## Introduction

In recent times, the most popular food-grade whitener, titanium dioxide (TiO_2_), has come under safety scrutiny, necessitating an alternative food whitening agent. TiO_2_ is also referred to as E171 when used as a food additive. The European Food Safety Authority announced in 2021 that E171 is to be considered no longer safe as a food additive, and as a result, the European Commission banned the use of the food additive E171 beginning in 2022^1^. TiO_2_ is a white, odorless powder that is often added to lighten or create a cloudy effect, as well as to provide opacity and whiteness for a variety of purposes. For this reason, it is currently the most effective white color used in many food applications. TiO_2_ can be found in a number of food products, including chewing gum, sweets, sauces, and baked goods^2,3^. However, there have been rising concerns about the genotoxicity of TiO_2_ nanoparticles and its potential impact on human health^4^. As a result, researchers need to find a replacement for TiO_2_ because E171 continues to face scrutiny about its safety, and consumers are increasingly demanding clean ingredients.

The rising consumer demand for high-protein dairy products has led to an increase in Greek-style yogurt production, resulting in the generation of high volumes of acid whey. A consumer study showed that the exponential rise in Greek yogurt production between 2007 and 2015 resulted in the generation of 1.5 million metric tons of acid whey in 2015^5^. Acid whey (AW) is the yellowish green liquid portion removed during Greek yogurt production. The liquid AW component is characterized by significant amounts of lactose, lipids, minerals, vitamins, proteins, and peptides^6^. Even though AW is a nutritious byproduct, its composition challenges further processing, limiting its utilization. AW has a low pH (< 4.5) and high lactic acid, which causes stickiness during drying, and the high mineral content in AW results in extensive fouling of the processing equipment^7^. Additionally, AW has a high biological oxygen demand making it challenging to dispose of into the environment without costly effects on the surrounding ecosystems^8^. Currently, AW is being utilized as a fertilizer, incorporated into animal feed, and converted into biofuel^5^. Dairy manufacturers often treat AW on-site as a waste stream and this requires filtration steps before disposal, which could be expensive and waste of a plentiful, nutritious byproduct. According to the United Nations, reducing food loss and food waste across food systems contributes to creating a more sustainable and resilient food system^9^. One of such ways is by upcycling food waste which involves transforming food waste into new, value-added products that can be used in various applications. Therefore, by upcycling food waste such as acid whey, into a valuable product, the dairy industry can effectively deal with the waste problem it currently faces.

Millets are highly nutritious cereal grains and a staple food for most people in the world’s arid and semiarid tropical areas. They are commonly cultivated in Asian and African countries and parts of Europe^10^. Millets are climate resilient, grow in adverse environmental conditions with almost no input, withstand unpredictable climate variations, and have a short growing season compared to other major cereal^11^^.^ Millets are rich in valuable nutrients (carbohydrates, proteins, dietary fibers, minerals, and vitamins) and contain significant amounts of amino acids, minerals, and phytochemicals that are related to numerous health benefits^12,13^. However, millets also have antinutrients such as phytates, tannins, and trypsin inhibitors that reduce their nutritional value and bioavailability^12^. Millets are nutritionally comparable or superior to other more commonly consumed cereals like maize, rice, and wheat. They provide a source of nutrients to the poor where the need for such is in high demand^14^. Despite being a superior seed grain in terms of nutrition, millet is still underutilized in developed and developing countries due to a lack of awareness. In Africa and Asia, millets are mainly consumed as a traditional staple food, and most western countries use millet primarily for animal feed^15^. Due to rising global hunger numbers, food security, and food system challenges, millets have gained popularity as a nutritious and sustainable crop alternative. Considering their high nutritional content and climate resiliency, millets are ideal for further processing into value-added nutritious functional products that can be incorporated into mainstream food production^16^.

Food powders have broad applications in food processing and are generally obtained from agricultural raw materials through different drying processes^17^. Spray drying is an economical, flexible, and efficient technique for creating dry powders from liquids or emulsions^18^. Because of its composition, spray drying AW is challenging and results in poor processing conditions and the stickiness of the spray dried powders. In spray drying operations, carrier materials mainly made from carbohydrate polymers, proteins, and lipids are used to improve the quality of the spray dried powder. These materials have good carrier properties, such as high molecular weight, high solubility, and high glass transition temperatures that prevent the stickiness of the powders^19^. According to Bylund^20^, cereals could be a good carrier material for AW because of their neutralizing effect on the pH of AW, which would improve the efficiency of the spray drying process. The use of millet as a carrier material could be a natural alternative to producing spray dried AW powders with enhanced functional and nutritional properties.

**Figure 1:**
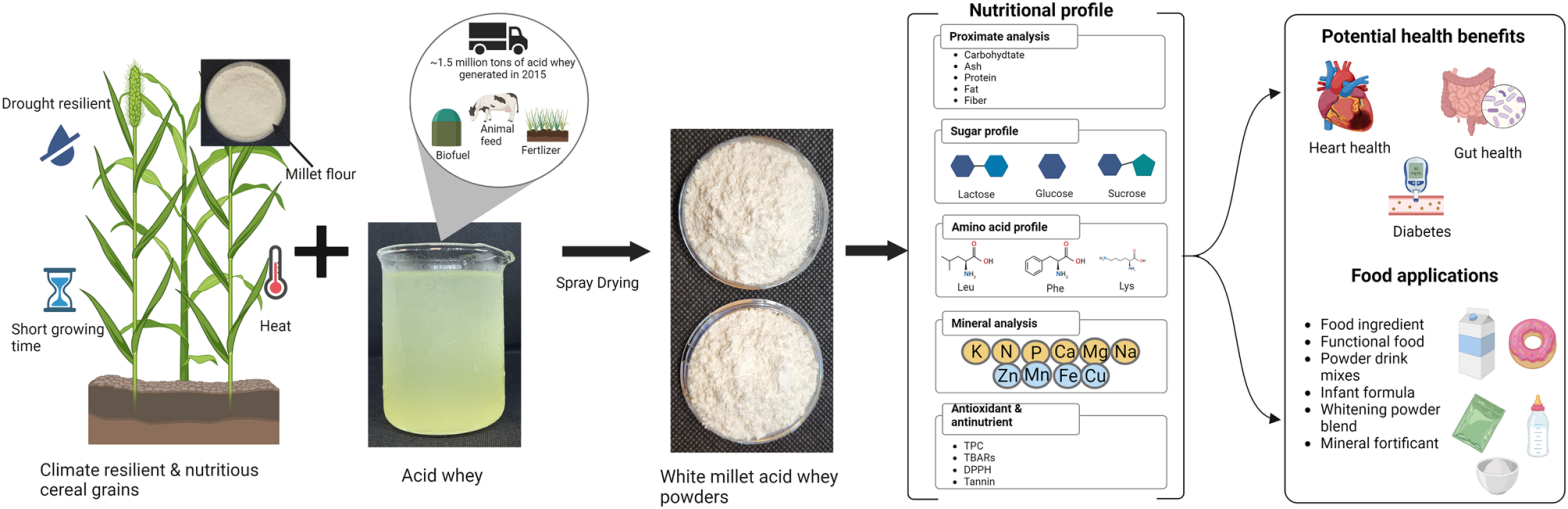
Overall summary of novel white powders and potential industrial applications contributing to sustainable food systems.

In previous studies^21^, we used Barnyard millet (*Echinochloa* species) and Little millet (*Panicum* species) flours as carrier materials to control and neutralize the lactic acid concentrations in AW and determined the physical properties of the spray dried powders. The resulting powders were white, with improved crystallization properties and enhanced powder yields. The encapsulating effect of millet on AW was attributed to the molecular interactions between the proteins and the particle surface in the acid whey millet matrix (AWM) which resulted in the production of free-flowing powders with reduced stickiness, low hygroscopicity, and increased yield^21^. Figure 1. illustrates the production of AWM millet powders, potential industrial applications of the powder and its contribution to sustainable food production. The aim of this study was to assess the color and nutritional properties of the novel AWM powders. The influence of spray drying on the color properties and nutrient content of the AWM powders was evaluated by comparing the powders to plain millet flours and spray dried millet soluble fractions.

## Materials and methods

### Materials

Commercially available Barnyard and Little millet grains were purchased from Manna foods (Chennai, India). A Butterfly Matchless 750-W mixer grinder was used for milling the grains. In-house yogurt culture obtained from FEAST Lab at the University of Missouri (Columbia, MO) was used for Greek yogurt fermentation. Whole milk was obtained from local grocery stores. A mesh was used for sieving the flour to obtain particles with a diameter under 500 µm.

### Chemicals and reagents

Folin-Ciocalteu and Thiobarbituric acid (TBA) were obtained from Sigma-Aldrich Corp. (St. Louis, MO). Acetic acid, gallic acid, and 2,2-diphenyl-1-picrylhydrazyl (DPPH) were purchased from Acros Organics (Morris Plains, NJ). Ethanol was purchased from Thermo Fisher Scientific (Hampton, NH). Sodium bicarbonate was purchased from Duda Energy LLC (Decatur, AL).

### Selection of AWM concentration

The selection of the optimal AWM concentration of 25% was based on a preliminary 2 × 5 full factorial analysis, which investigated the interaction effects of the type of millet (Little and Barnyard) and the concentration of AWM solution (10%, 25%, 50%, 75% and 100%) on response variables such as pH, powder yield, color, water activity, moisture content, density, and flowability. Results indicated that the 10% and 25% AWM concentrations yielded the desired powder outcome, prompting a subsequent study^21^ to further characterize their physical and functional properties. The results showed that the 25% AWM concentration was superior in terms of color, glass transition temperature and particle morphology, indicating a more free-flowing powder.

### Preparation of acid whey and feed solution for spray drying

Greek yogurt was produced based on the in-house lab protocol for yogurt production. To prepare Greek yogurt, whole milk (4 L) was heated to 82–85 °C with constant stirring. After cooling the milk base to 40–45 °C, the starter culture was added. The mixture was allowed to ferment in an incubator at 42 °C for 6 to 10 h for coagulation. Once fermentation was complete the yogurt was centrifuged at 5000 rpm for 20 min to separate the solid components from the liquid and obtain AW. The cooled AW was decanted and stored at 4 °C for further analysis. The total volume of acid whey obtained from producing 4 L of was ~ 2.2–2.7 L. To prepare the AWM feed solution, Barnyard, and Little millet flours were suspended in AW to obtain a concentration of 25% w/v. The suspension was stirred for 10 min at 200 rpm, to increase the solubilization of proteins^22^. To prepare the millet soluble fractions, 25% w/v of millet was suspended in distilled water and homogenized for 10 min at 3000 rpm. Then the solution was allowed to stand for 30 min–1 h to enable decanting of the supernatant. The AWM solution was vacuum filtered to obtain the soluble fraction for spray drying. The description for each sample formulation is given in Table 1.

**Table 1:** AWM formulation and sample code description.

Sample	Millet flour (g)	Acid whey (ml)	Distilled water (ml)	Concentration % w/v
BY (plain Barnyard millet flour)	25	–	–	–
LT (plain Little millet flour)	25	–	–	–
SD_BY (spray dried Barnyard millet soluble fraction)	25	–	100	25
SD_LT (spray dried Little millet soluble fraction)	25	–	100	25
BAW (Barnyard millet + acid whey)	25	100	–	25
LAW (Little millet + acid whey)	25	100	–	25

### Microencapsulation by spray drying

A laboratory-scale spray-dryer (Büchi B290 mini spray-dryer, Flawil, Switzerland) equipped with a 0.7 mm diameter and a peristaltic feed pump was used to spray dry the AWM solution. The input temperature, aspirator, feed pump rate, and gas flow rate were 160 °C, 90%, 15%, and 45%, respectively. These parameters were selected based on previously optimized parameters from prior experiments^21^. The obtained powders were stored at 4 °C in sealed glass jars until further analysis.

### Color

The color characteristics of the spray dried powder were determined using a handheld chromameter (Konica Minolta CR-410, Chiyoda, Tokyo, Japan). The values were expressed in L* (brightness/darkness), a* (redness/greenness), and b*(blueness/yellowness) coordinates and used to calculate the color difference (∆E), chroma (C*), hue angle (°) and Whiteness index of the powders according to Eqs. (1, 2, 3 and 4). The color difference was calculated as the difference between the spray dried powders and TiO_2_.

1 $$\Delta E= \sqrt{{\left({L}_{1}^{*}-{L}^{*}\right)}^{2}+{({a}_{1}^{*}-{a}^{*})}^{2}+{({b}_{1}^{*}-{b}^{*})}^{2}}$$

2 $$\mathrm{Chroma }(\mathrm{C}*) = \sqrt{({a}^{*2})+{(b}^{*2})}$$

3 $$\mathrm{Hue\,angle }(\mathrm{h}*) = {tan}^{-1}(\frac{b*}{a*})$$

4 $$\mathrm{Whiteness\,Index }(\mathrm{WI}) =100 - \sqrt{{\left(100-{L}^{*}\right)}^{2}+{a}^{*2}+{b}^{*2}}$$

### Chemical analysis

All spray dried powder and millet flour samples were sent to the University of Missouri Agricultural Experiment Station Chemical Laboratory (Columbia, MO) for proximate, amino acid, and sugar profiles. Samples for mineral analysis were sent to the Soil and Plant Testing Laboratory at the University of Missouri Columbia.

### Proximate analysis

Powder samples were analyzed for nitrogen following the Kjeldahl AOAC method 984.13^23^, and crude protein value calculated by multiplying the percent nitrogen by 6.25 (Crude protein = %N $\times$ 6.25). Moisture content was determined by drying 2 g of the powder samples in a vacuum oven at 105 °C and calculating the weight loss upon drying^24^. Ash content was determined by heating powder samples to 600 °C for 2 h according to AOAC method 942.05^25^. The crude fiber content was determined following AOAC method 978.10^26^. Briefly, the powder sample (2 g) was immersed in lightly boiling 200 ml 0.128 M H_2_SO_4_ for 30 min. Then the boiled sample was filtered to remove the acid solution and then boiled in 200 ml 0.313 M NaOH for 30 min. After being treated, the filtrate was rinsed with boiling water and then dried at 230 °C for 2 h. The dried sample was ashed in a furnace at 550 °C for 4 h and weighed. Crude fiber was determined as the residue remaining after acid and alkaline digestions. Crude fat content was determined by acid hydrolysis according to AOAC method 954.02^27^. Carbohydrate content was determined by calculating the difference (100 – sum of moisture, crude protein, ash, crude fat and crude fiber). The proximate composition was expressed in grams per 100 g of dry basis. For comparison, all these determinations were also carried out on plain Barnyard and Little millet flours.

### Amino acid analysis

Amino acid analysis was performed using cation-exchange chromatography (cIEC-HPLC) coupled with post-column ninhydrin derivatization and quantitation according to the AOAC Official Method 982.30^28^. Acid hydrolysis of sample was done with 6 N HCL at 110 $^\circ$ C for 24 h to break down proteins into individual amino acid. NaOH solution was used to neutralize the solution and the hydrolysate was then filtered through a 0.45-μm to remove any particulate matter. Then the filtered hydrolysate was then injected into the cIEC-HPLC column. Then the amino acids were derivatized with ninhydrin reagent in a post-column reactor and detected using an ultraviolet detector and the signal intensity was recorded. The amino acids were quantified by comparing the signal intensity to a standard curve generated from known standards of individual amino acids.

Methionine and cysteine were analyzed after cold performic acid oxidation using 2 mL performic acid overnight at 0–5 °C before acid hydrolysis. The digested samples were then quantified by cation-exchange liquid chromatography followed by column ninhydrin derivatization for UV/Vis detection. Tryptophan was determined separately by alkaline hydrolysis followed by cation exchange chromatography according to the AOAC Official Method 988.15^29^.

### Sugar profile analysis

The sugar profile (glucose, fructose, sucrose, lactose, maltose, raffinose, stachyose, and verbascose) was determined by chromatographic methods according to Churms et al.^30^ and Honda et al.^31^^.^ Individual sugar profiles were analyzed using HPLC system coupled with a refractive index detector. The sample extracts were prepared using 80% ethanol and then filtered through a 0.45 µm membrane filter to remove any particulate matter. Analysis was done using a Bio-Rad HPX 300 × 7.8 mm LC column at 80 °C. The mobile phase was composed of acetonitrile and purified water and a flow rate of 0.7 mL/min. The identification standards used include d-glucose, fructose, sucrose, lactose, maltose, raffinose, stachyose, and verbascose.

### Mineral analysis

The spray dried powders and millet flours were analyzed for minerals at the Soil and Plant Testing laboratory, University of Missouri using Inductively Coupled Plasma—Optical Emission Spectroscopy (ICP-OES) techniques according to the procedures described in AOAC Official Method 985.01^32^. Powder samples were digested by concentrated hydrochloric acid and then filtered to remove any particulate matter. Calibration standards were prepared by diluting multi element standard solutions containing the mineral of interest (N, P, K, Ca, Mg, Zn, Fe, Mn, Cu, Na). The results were calculated based on the calibration curve and expressed in mg/kg or ppm.

## Antioxidant and antinutritional properties

### Preparation of extracts

The ethanolic extracts of the samples were prepared according to the method described by Malik et al.^33^ In brief, about 1 g of each sample was extracted with 10 mL of 80% ethanol at 25 °C for 24 h in a shaker. Afterwards, the slurry was centrifuged (5000 rpm, 20 min) and the supernatant collected and dried for 24 h at room temperature. Before analysis, the extracts were reconstituted with 5 mL of distilled water.

### Total phenolic content (TPC)

The TPC of the AWM powder samples and the millet flour samples were measured according to the Folin–Ciocalteu reagent method described by Singleton et al.^34^ with minor modifications. About 2 mL of extract was mixed with 1 mL distilled water, 0.5 mL Folin–Ciocalteu reagent, and 2 mL of 20% sodium bicarbonate. After incubation in a water bath at 40 °C 25 min, the TPC was determined using a UV–visible spectrophotometer, and absorbance was taken at 765 nm. The results were expressed in milligrams of gallic acid equivalents per gram of dry powder (mg GAE/g).

### DPPH radical scavenging activity

The antioxidant activity of the sample extracts was measured by the DPPH radical scavenging method described by Horvat et al.^35^^.^ The assay was prepared by mixing 0.2 mL of sample extract with 1 mL 0.5mMol/L 2,2-diphenyl-1-picrylhydrazyl (DPPH) solution and 2 mL ethanol. The mixture was incubated in a dark place at room temperature for 30 min. The absorbance of the reaction mixtures was measured at 517 nm. The percentage of inhibition of free radical DPPH (decrease in absorbance) was calculated against the blank (DPPH + ethanol) using Eq. (5).

5 $$\mathrm{\% DPPH\,inhibition }= \frac{{A}_{0}-{A}_{1}}{{A}_{0}}\times 100$$

where A_0_ is the absorbance of the blank sample at time = 0 and A_1_ is the absorbance of the analyzed sample after 30 min.

### Estimation of thiobarbituric acid reactive substances (TBARS)

TBARS was calculated and measured as previously described by Zeb et al.^36^ In brief, a 4.0 mM standard solution of TBA was prepared by dissolving 57.66 mg of TBA in 100 ml glacial acetic acid. The spray dried powders and millet flour sample extract (1 mL) was mixed with 1 mL TBA reagent and heated in a water bath at 95 °C for 1 h. The mixture was cooled to room temperature, and the absorbance was measured at 532 nm. Malondialdehyde tetrabutylammonium (MDA) salt was used to develop the standard curve at 0.1, 0.2, 0.4, 0.6, and 0.8 mM MDA concentrations. The concentrations of TBARS were calculated using Eq. (6).

6 $$\mathrm{TBARS }(\mathrm{\mu M}/\mathrm{g}) =\frac{Ac \times V}{W}$$

where Ac is the concentration determined from the calibration curve, W is the weight of the sample, and V is the volume in mL of the total extract prepared.

### Estimation of tannin content

The total condensed tannin content was estimated using the vanillin-HCL method as outlined by Burns^37^. In brief, about 0.5 mL of 10 mg/mL of the sample extract was combined with 3 mL of 4% w/v vanillin and 1.5 mL of hydrochloric acid, followed by mixing. The mixture was left to stand for 15 min at room temperature and the absorbance was measured at 500 nm with a UV/Vis spectrophotometer. This analysis was done in triplicate. Catechin was used for the standard curve, and tannin content was expressed as mg catechin equivalents/g of dried extract (mg CE/g).

### Statistical Analysis

All antioxidant and antinutrient experiments were done in triplicate using three independent powder samples. Analysis of variance (ANOVA) of mean values was carried out using the JMP 14.0 statistical software program (SAS Institute Inc, Cary, NC). Means were compared using the Tukey test, and results with a confidence level of 95% was accepted as significant (*p* < 0.05). Results were presented as mean ± standard deviation.

## Results and discussion

### Color

**Figure 2:**
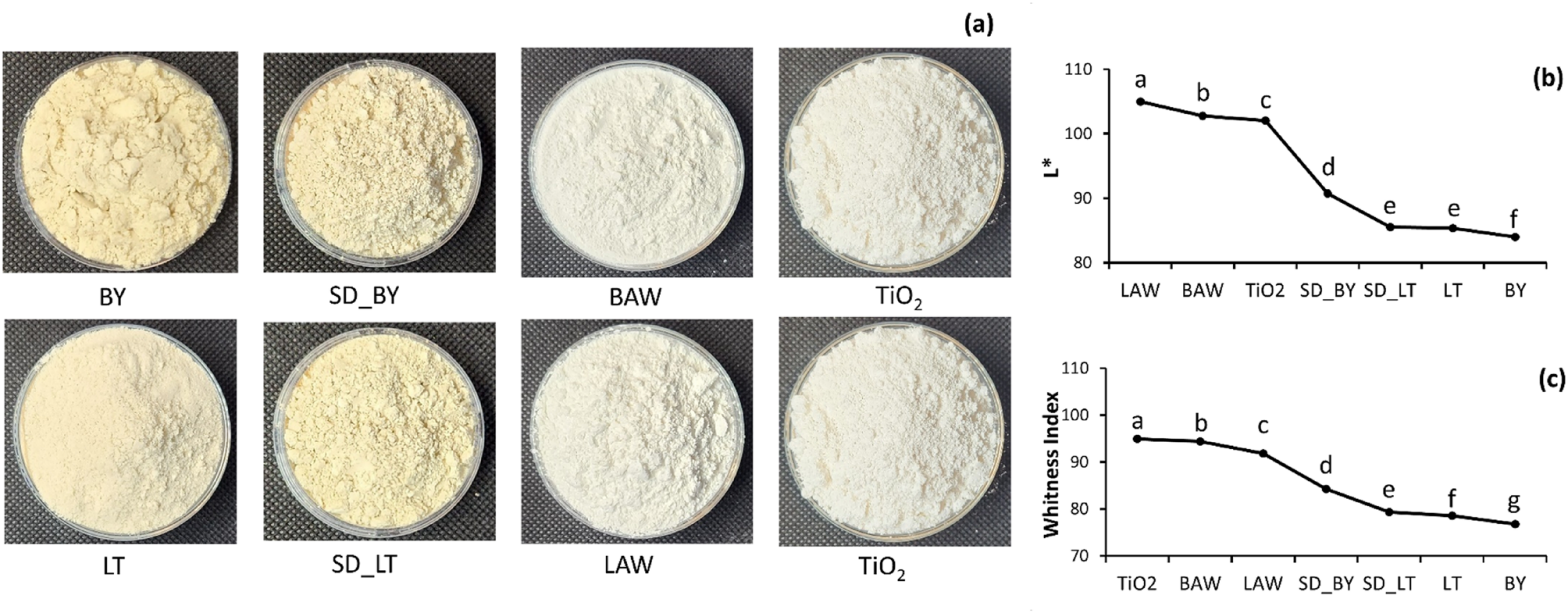
(**a**) millet flour and spray dried samples compared to TiO_2_ (**b**) Lightness (L*) and (**c**) witnesses index values of the samples. Mean values followed by a different letter are significantly different. Legend: Image, L*, and whiteness index values of the flour and powder samples. BY: plain Barnyard millet flour; LT: plain Little millet flour; SD_BY: spray dried Barnyard millet soluble fraction; SD_LT: spray dried Little millet soluble fraction; BAW: 25% w/v Barnyard millet + acid whey; LAW: 25% w/v Little millet + acid whey.

The visual appearance of the millet flour (BY, LT), spray dried millet fraction (SD_BY, SD_LT), and AWM powders (BAW, LAW) compared to TiO_2_ is shown in Fig. 2a. The millet flour and spray dried millet flour were light to creamy in color and were significantly (*p* < 0.05) less white than the AWM powders. Visually no difference was observed between the two AWM powders formulations and TiO_2_. The complete color attributes of the flour and spray dried powder samples are shown in Table 2. The sample’s color difference (∆E) was determined by comparing the sample’s color attributes to those of TiO_2_. A higher ∆E indicates a greater difference in color between the sample and TiO_2_. The AWM powder samples had significantly lower $\Delta$ E values compared to the other samples, with BAW having the least color difference. The addition of acid whey to millet significantly (*p* < 0.05) improved the whiteness index of the AWM powders. As seen in Fig. 2b and c, the lightness and whiteness index values of the AWM powders were closer to TiO_2_ values. Though LAW powders had significantly higher Lightness (L*) values than BAW, BAW powders had a significantly higher whiteness index value. It is also important to note that the addition of millet significantly improved the luminosity (L*) of the powders. The b* values associated with the creamy color from the millet flours were decreased in the AWM powders. Similar L*, b*, and whiteness index values were reported for plain AW and AWM powders^21^. According to these results, AW encapsulated with millet improved powder functionality while retaining whiteness.

**Table 2:** Color parameters of millet flour and spray dried powder samples.

Color parameters	BY	SD_BY	BAW	LT	SD_LT	LAW
L*	83.98 $\pm$ 0.01^a^	90.73 $\pm$ 0.05^b^	102.78 $\pm$ 0.09^c^	85.37 $\pm$ 0.02^d^	85.53 $\pm$ 0.11^d^	105.01 $\pm$ 0.12^e^
a*	1.35 $\pm$ 0.01^a^	1.01 $\pm$ 0.01^b^	− 0.54 $\pm$ 0.01^c^	0.83 $\pm$ 0.01^d^	1.12 $\pm$ 0.01^e^	− 0.31 $\pm$ 0.00^f^
b*	16.83 $\pm$ 0.02^a^	12.73 $\pm$ 0.02^b^	4.81 $\pm$ 0.01^c^	15.68 $\pm$ 0.01^d^	14.72 $\pm$ 0.02^e^	6.43 $\pm$ 0.12^f^
Chroma	16.88 $\pm$ 0.02^a^	12.76 $\pm$ 0.03^b^	4.84 $\pm$ 0.02^c^	15.70 $\pm$ 0.02^d^	14.76 $\pm$ 0.02^e^	6.44 $\pm$ 0.13^f^
Hue angle (°)	85.40 $\pm$ 0.02^a^	85.47 $\pm$ 0.04^a^	− 83.54 $\pm$ 0.09^b^	86.95 $\pm$ 0.03^c^	85.63 $\pm$ 0.06^a^	− 87.22 $\pm$ 0.05^d^
$\Delta$ E	21.79 $\pm$ 0.02^a^	13.89 $\pm$ 0.05^b^	1.33 $\pm$ 0.05^c^	19.98 $\pm$ 0.01^d^	19.34 $\pm$ 0.09^e^	3.60 $\pm$ 0.16^f^
Whiteness index (WI)	76.73 $\pm$ 0.02^a^	84.23 $\pm$ 0.04^b^	94.42 $\pm$ 0.05^c^	78.54 $\pm$ 0.01^d^	79.33 $\pm$ 0.08^e^	91.84 $\pm$ 0.17^f^

Means having different letters as superscripts within the same row differ significantly (n = 5) at *p* < 0.05.

Color parameters of the powder samples. BY: plain Barnyard millet flour; LT: plain Little millet flour; SD_BY: spray dried Barnyard millet soluble fraction; SD_LT: spray dried Little millet soluble fraction; BAW: 25% w/v Barnyard millet + acid whey; LAW: 25% w/v Little millet + acid whey.

### Proximate analysis

**Figure 3:**
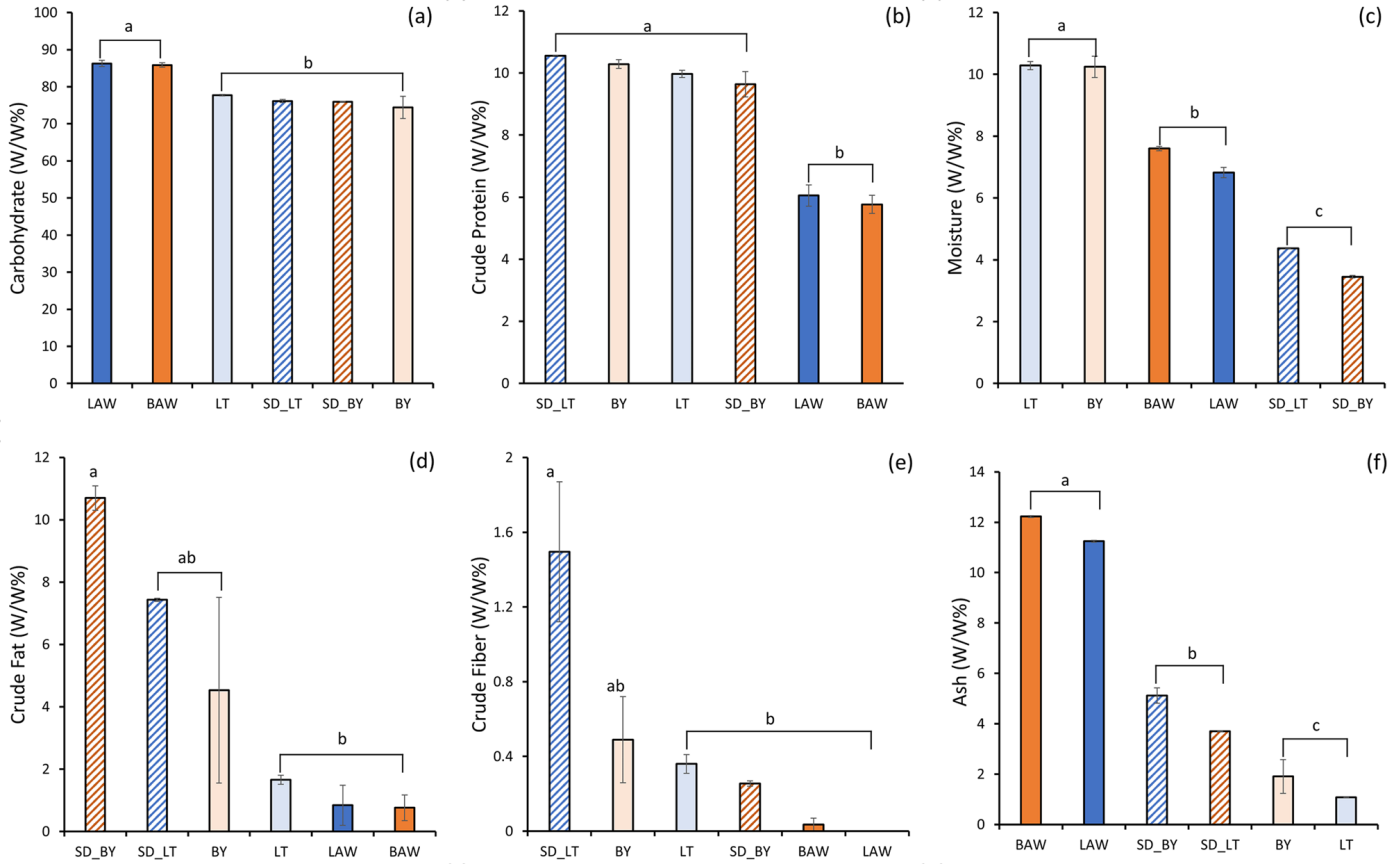
Proximate analysis of millet flours and acid whey millet powders (**a**) carbohydrate (**b**) crude protein (**c**) moisture (**d**) crude fat (**e**) crude fiber (**f**) ash. Mean values followed by different letters are significantly different (*p* < 0.05). Legend: Proximate analysis of powder samples. BY: plain Barnyard millet flour; LT: plain Little millet flour; SD_BY: spray dried Barnyard millet soluble fraction; SD_LT: spray dried Little millet soluble fraction; BAW: 25% w/v Barnyard millet + acid whey; LAW: 25% w/v Little millet + acid whey.

The results of the proximate compositions of BY, LT, SD_BY, SD_LT, BAW and LAW are presented in Fig. 3. The carbohydrate, crude protein, and lipid content of the millet flours were compared to already reported values for millet flour^38^. In this study, the carbohydrate content of BY and LT was higher by 13.7% and 16% respectively, compared to the previously reported values^38^. The significantly (*p* < 0.05) higher carbohydrate content in the AWM powders is due to the high lactose content in acid whey. The crude protein content of BY and LT was higher by 66% and 29.5%, respectively, compared to the previously reported values^38^. The crude protein content in the AWM powders was significantly (*p* < 0.05) lower when compared to the crude protein content in millet flours. However, when compared to crude protein results reported for plain AW powders (1.71 to 3.71 mg/g), the AWM powder from this study had a higher crude protein content^8^. There was no significant difference between the crude protein values of the plain millet flour and the spray dried millet fraction. This indicates that the spray drying process did not significantly affect the protein content of the spray dried powders. The lower crude protein value in the AWM spray dried powder samples could be attributed to the concentration of millet in the AWM feed solution. The crude protein values for BY and LT are consistent with other reports that showed higher protein content in some millet varieties when compared to rice and maize^12^. AWM powders had a much lower moisture content than millet flour due to the spray drying process. However, the moisture content was significantly lower in the spray dried millet fraction. This is due to the high acid content in acid whey, resulting in a more hygroscopic powder. The crude fat content in millet flours was present in significantly lower amounts when compared to already reported values for Barnyard and Little millet flours. The fat content was also lower in the AWM powders. The decrease in crude fat in the AWM powders could be attributed to the oxidation and degradation that occurs as a result of increasing the surface area of the soluble fractions during spray drying^39^. The concentration of crude fiber for the millet flours was much lower in this case than in already reported values. This could be due to the difference in grain variety and processing conditions.

Very little crude fiber was present in the AWM spray dried powders, which could be due to the removal of insoluble fiber (mostly from the seed coats) while filtering the feed solution. When compared with millet flour, the AWM samples had a significantly (*p* < 0.05) higher ash content. The higher ash concentration of the AWM powder could be attributed to the high mineral content present in acid whey. These results are in line with the ash content reported for whey powders^40^, which observed 12.93% ash content for acid whey powders. It was reported that the main minerals present in AW are K, Ca, P, with lower concentrations of Mg and Na^6^. Significantly higher ash content was observed in the spray dried millet fractions than in the millet flour. This could be due to the solubilization of certain minerals in the millet flour during the preparation of the soluble fraction for spray drying.

### Sugar profile

Table 3 presents the amounts of eight sugars (fructose, glucose, sucrose, lactose, maltose, raffinose, stachyose, and verbascose) present in the spray dried AWM powders and millet flour samples. There was an increase in most of the sugars in the spray dried millet fractions when compared to the millet flour, which could be due to the high concentration of millet soluble fraction in the solution as a result of the preparation process that aids the solubilization of millet solids. The AWM powder contained significantly higher glucose and lactose when compared to the other millet samples. This result was expected due to the high lactose content in the fermented plain AW. Lactose is a reducing disaccharide composed of glucose and galactose. It constitutes the major carbohydrate in AW^41^ so it was expected that the glucose and raffinose content in the AWM powder to increase. Similar amounts of sucrose, glucose, maltose, and fructose were found in rice compared to plain millet flour^42^.

**Table 3:** Sugar profile of millet flours and acid whey millet powders.

Sugar	BY (%)	LT (%)	SD_BY (%)	SD_LT (%)	BAW (%)	LAW (%)
Fructose	0.16 $\pm 0.08$^a^	0.02 $\pm 0.02$^a^	1.27 $\pm$ 0.00^a^	0.63 $\pm 0.00$^a^	0.80 $\pm 0.67$^a^	0.46 $\pm 0.37$^a^
Glucose	0.31 $\pm 0.01$^a^	0.24 $\pm 0.00$^a^	1.83 $\pm$ 0.00^b^	1.50 $\pm 0.00$^b^	4.40 $\pm 0.32$^c^	4.37 $\pm 0.06$^c^
Sucrose	0.30 $\pm 0.01$^a^	0.32 $\pm 0.00$^a^	1.15 $\pm 0.00$^b^	0.85 $\pm 0.00$^b^	1.04 $\pm 0.19$^b^	1.09 $\pm 0.11$^b^
Lactose	0.61 $\pm 0.39$^a^	0.24 $\pm 0.24$^a^	0.08 $\pm 0.00$^a^	0.22 $\pm 0.00$^a^	52.71 $\pm 0.60$^b^	52.91 $\pm 3.01$^b^
Maltose	0.06 $\pm 0.03$^a^	0.20 $\pm 0.08$^a^	$1.00\pm 0.00$^b^	1.76 $\pm 0.00$^c^	0.20 $\pm 0.04$^a^	0.05 $\pm$ 0.05^a^
Raffinose	0.07 $\pm 0.02$^a^	0.07 $\pm 0.02$^a^	0.31 $\pm 0.00$^c^	0.22 $\pm 0.00$^b^	0.34 $\pm 0.02$^c^	0.31 $\pm 0.01$^c^
Stachyose	0.00 $\pm 0.00$^a^	0.00 $\pm 0.00$^b^	0.30 $\pm 0.00$^c^	0.16 $\pm 0.00$^d^	0.11 $\pm 0.03$^d^	0.01 $\pm 0.01$^a^
Verbascose	0.00 $\pm 0.00$	0.00 $\pm 0.00$	0.00 $\pm 0.00$	0.00 $\pm 0.00$	0.00 $\pm 0.00$	0.00 $\pm 0.00$

Means having different letters as superscripts within the same row differ significantly at *p* < 0.05.

Sugar profile of powder samples. BY: plain Barnyard millet flour; LT: plain Little millet flour; SD_BY: spray dried Barnyard millet soluble fraction; SD_LT: spray dried Little millet soluble fraction; BAW: 25% w/v Barnyard millet + acid whey; LAW: 25% w/v Little millet + acid whey.

### Amino acid composition

**Figure 4:**
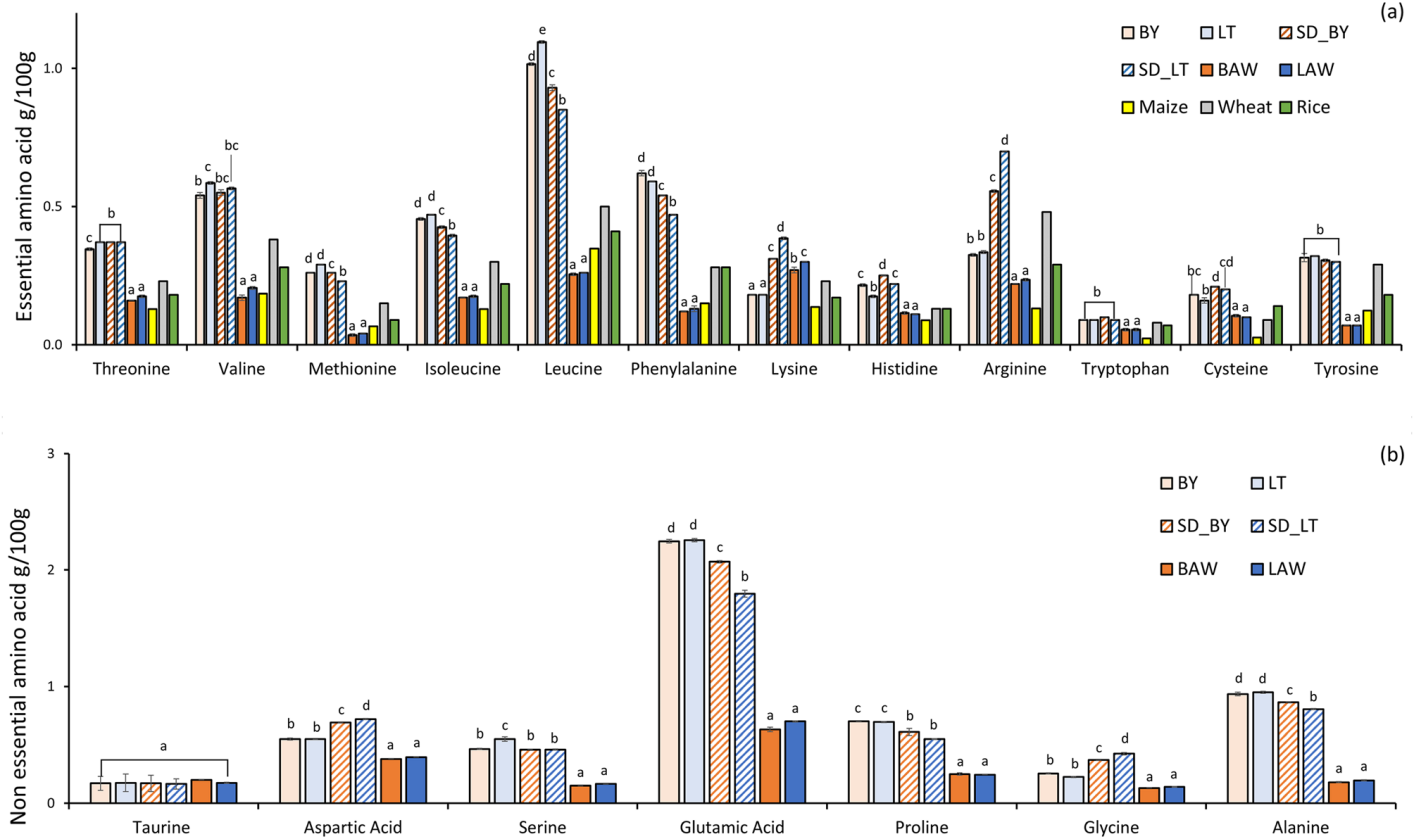
Amino acid profile of AWM sample and millet flour samples compared to popular cereal grains. W/W% = grams per 100 g of sample. The data for maize, wheat, and rice was obtained from Chandra et al.^38^. Mean values followed by a different letter are significantly different. Legend: Amino acid profile of powder samples. BY: plain Barnyard millet flour; LT: plain Little millet flour; SD_BY: spray dried Barnyard millet soluble fraction; SD_LT: spray dried Little millet soluble fraction; BAW: 25% w/v Barnyard millet + acid whey; LAW: 25% w/v Little millet + acid whey.

The amino acid profiles of AWM powders and pure millet flour compared to maize, wheat and rice is illustrated in Fig. 4. Essential amino acids are crucial building blocks of bodily proteins that must be supplied through the diet as they cannot be synthesized by the human body^43^. The millet flours and spray dried millet fractions were significantly (*p* < 0.05) higher in all the essential amino acids than the AWM powders. This could be attributed to the concentration of millet in the feed solution and the denaturation of protein molecules during processing and spray drying. Notably, the AWM powders had a high lysine content, which could be attributed to the high lysine content in AW. This finding is supported by a previous study that showed high lysine content in deproteinized whey powders^44^. Leucine was the most abundant essential amino acid in the millet flours, and the newly developed AWM powder also retained significant amounts of leucine. Leucine, isoleucine, and valine are branched-chain amino acids that are critical building blocks of bodily proteins and have also been shown to have therapeutic effects against some diseases when taken as supplements^45^. The presence of these branched-chain amino acids makes AWM powder an excellent ingredient for the development of functional foods that require these essential amino acids. Additionally, higher amounts of tryptophan and arginine were found in the plain millet samples and AWM powders than in maize. The presence of significant amounts of tyrosine, glycine, glutamine, arginine, proline, and taurine, which are conditionally essential amino acids, indicates that AWM powder could also contribute to the nutritional quality of foods developed for specific health conditions such as pregnancy, lactation, and critical illness^48^. Based on these results, using AWM powder in food formulation could help reduce food waste while providing consumers with high-nutrient, functional food options.

### Mineral analysis

**Figure 5:**
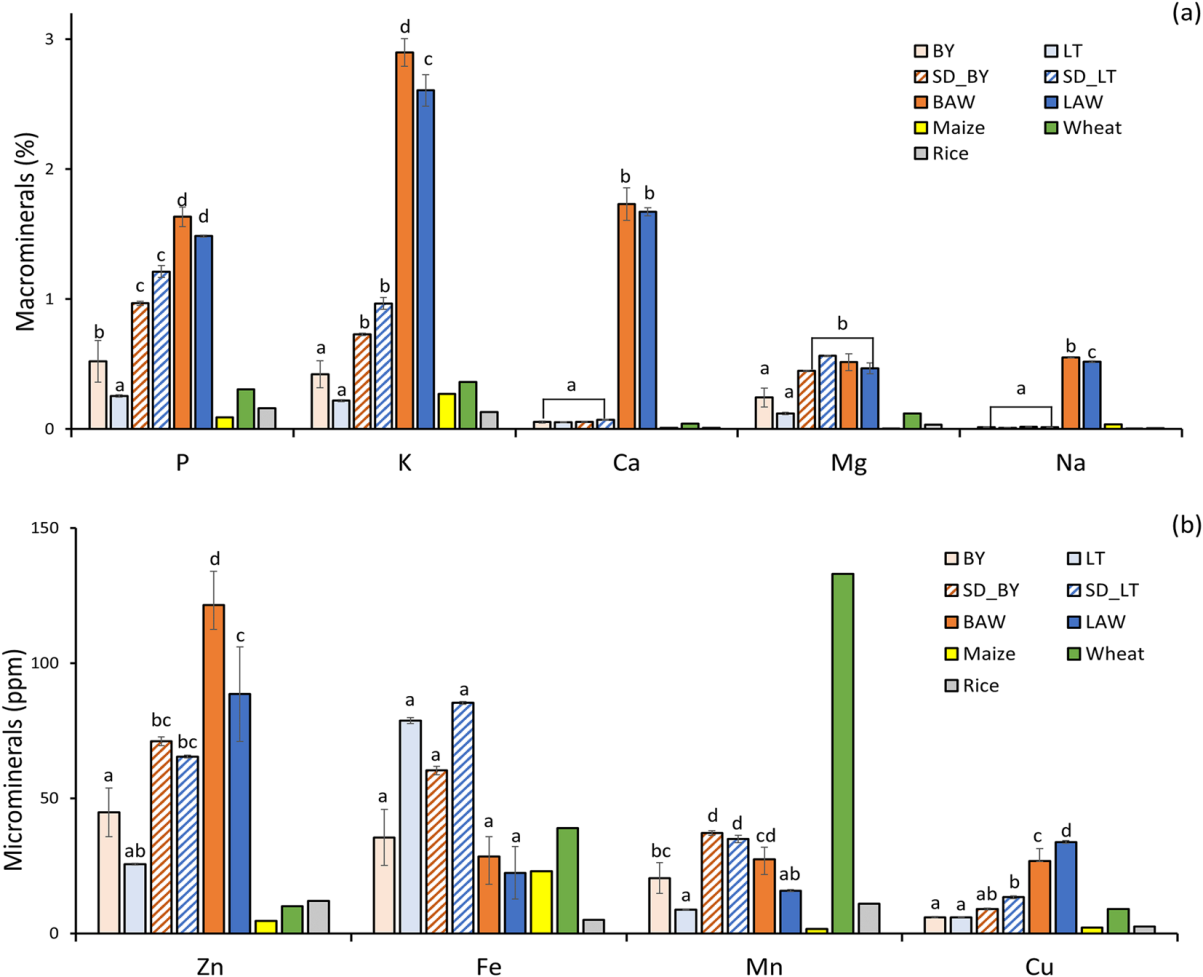
Mineral profile of acid whey millet powders and millet flours (**a**) macronutrients (**b**) micronutrients. The data for maize, wheat, and rice was obtained from Chandra et al.^38^. Legend: Mineral content in the powder samples .BY: plain Barnyard millet flour; LT: plain Little millet flour; SD_BY: spray dried Barnyard millet soluble fraction; SD_LT: spray dried Little millet soluble fraction; BAW: 25% w/v Barnyard millet + acid whey; LAW: 25% w/v Little millet + acid whey.

The mineral composition of the AWM powder and the millet flour is shown in Fig. 5. Minerals are essential nutrients for maintaining and promoting human physical and mental health. They are divided into two main groups (macro minerals and microminerals) based on the amounts required to maintain good health. Macro minerals are needed in large amounts, while microminerals are needed in trace amounts, but both groups are very necessary for good health^47^. The AWM powders had significantly (*p* < 0.05) higher P, K, Ca, and Na values when compared to the soluble fractions (SD_BY and SD_LT) and liquid AW (Table 4). The increased mineral content in the AWM powder resulted from the concentration of the high amounts of minerals present in the liquid AW by the spray drying process. Similar results were reported for the mineral content of dry AW fractions^6^. Additionally, when compared to reported values for maize, wheat, and rice^38^, the P, K, Ca, Mg and Na content in the millet flour, spray dried millet fractions and AWM powders were much higher. The same trend was observed for the micromineral Zn in which the plain millet samples (BY, LT, SD_BY and SD_LT) and AWM powders had higher concentrations of Zn than already reported values for maize, wheat and rice. The AWM powders had significantly higher Zn and Cu when compared to the plain millet sample and liquid AW (Table 4). The decrease of Fe and Mg in the AWM powders could be attributed to the difference in the concentration of millet in the AWM and millet fractions feed solution. Also, bran loss during the processing of the feed solution could contribute to the decrease in Fe as most of the iron content in little millet can be found in the bran. The high iron content found in AWM formulations agrees with reported results of increased iron content (by 94%) with the addition of little millet flour to bread dough^48^. It is important to understand the nutritional quality of AWM powder’s mineral composition and how it can contribute to the development of new functional food products and ingredients.

**Table 4:** Mineral content comparison of millet flour, AWM powder samples, and liquid acid whey.

Mineral	BY	LT	SD_BY	SD_LT	BAW	LAW	AW
Ca w/w %	0.05 $\pm$ 0.01^a^	0.05 $\pm$ 0.00^a^	0.06 $\pm$ 0.00^a^	0.07 $\pm$ 0.00^a^	1.73 $\pm$ 0.12^b^	1.67 $\pm$ 0.03^b^	0.35
Mg w/w%	0.24 $\pm$ 0.07^a^	0.11 $\pm$ 0.00^a^	0.45 $\pm$ 0.01^b^	0.56 $\pm$ 0.01^b^	0.51 $\pm$ 0.06^b^	0.47 $\pm$ 0.04^b^	0.04
P	0.52 $\pm 0.16$ ^b^	0.25 $\pm 0.01$ ^a^	0.97 $\pm$ 0.02^c^	1.21 $\pm$ 0.05^c^	1.63 $\pm$ 0.08^d^	1.48 $\pm$ 0.01^d^	0.06
K	0.42 $\pm$ 0.10^a^	0.22 $\pm$ 0.01^a^	0.73 $\pm$ 0.01^b^	0.97 $\pm$ 0.05^b^	2.90 $\pm$ 0.11^d^	2.61 $\pm$ 0.12^c^	0.15
Na	0.01 $\pm$ 0.00^a^	0.01 $\pm$ 0.00^a^	0.02 $\pm$ 0.00^a^	0.02 $\pm$ 0.00a	0.55 $\pm$ 0.01^b^	0.52 $\pm$ 0.01^c^	0.04
Fe ppm	35.49 $\pm$ 10.38^a^	78.71 $\pm$ 1.11^a^	60.29 $\pm$ 1.54^a^	85.31 $\pm$ 0.51^a^	28.47 $\pm$ 07.35^a^	22.38 $\pm$ 9.69^a^	1.55
Zn ppm	44.80 $\pm$ 9.03^a^	25.62 $\pm$ 0.03^ab^	71.04 $\pm$ 1.59^bc^	65.43 $\pm$ 0.49^bc^	121.47 $\pm$ 12.51^d^	88.53 $\pm$ 17.53^c^	12.6
Cu ppm	5.91 $\pm$ 0.25^a^	5.93 $\pm$ 0.09^a^	9.02 $\pm$ 0.35^ab^	13.36 $\pm$ 0.57^b^	26.79 $\pm$ 4.60^c^	33.79 $\pm$ 0.56^d^	< 0.1
Mn ppm	20.45 $\pm$ 5.66^bc^	8.74 $\pm$ 1.80^a^	37.09 $\pm$ 0.83^d^	34.95 $\pm$ 1.36^d^	27.37 $\pm$ 4.50^ cd^	15.80 $\pm$ 0.40^ab^	0.12

Means having different letters as superscripts within the same row differs significantly at *p* < 0.05. <  = values are below detection limit. AW was not included in the significance test. The AW data for P, K, Na was obtained from Wong et al.^6^

Mineral content in the powder and liquid acid whey samples. BY: plain Barnyard millet flour; LT: plain Little millet flour; SD_BY: spray dried Barnyard millet soluble fraction; SD_LT: spray dried Little millet soluble fraction; BAW: 25% w/v Barnyard millet + acid whey; LAW: 25% w/v Little millet + acid whey; AW: liquid acid whey.

### Antioxidant and antinutritional properties

**Figure 6:**
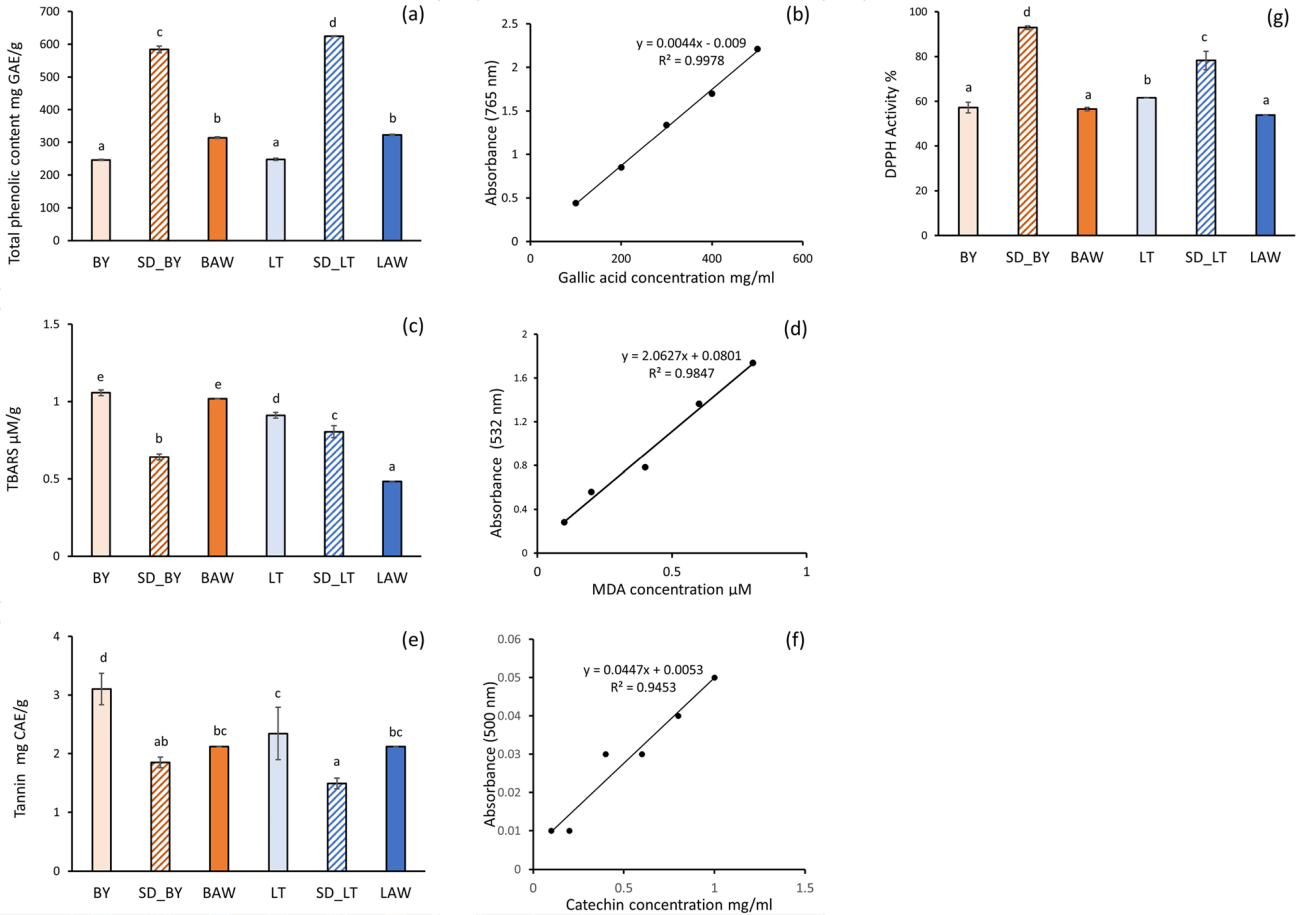
Antioxidant and antinutritional properties of AWM powders and millet flour (**a**) Total phenol content (**b**) Gallic acid calibration curve (**c**) TBARS concentration (**d**) MDA calibration curve (**e**) Tannin content (**f**) Catechin calibration curve (**g**) DPPH activity. Significant differences among samples are expressed by different lowercase letters. Legend: Antioxidant and antinutritional properties of the powder samples. BY: plain Barnyard millet flour; LT: plain Little millet flour; SD_BY: spray dried Barnyard millet soluble fraction; SD_LT: spray dried Little millet soluble fraction; BAW: 25% w/v Barnyard millet + acid whey; LAW: 25% w/v Little millet + acid whey.

The content and tannin content of the millet and AWM powder samples are given in Fig. 6. The TPC was significantly higher in the spray dried millet fractions compared to the millet flour. This could be attributed to the solubilization of phenolic compounds during the preparation of millet soluble fractions for spraying, which could increase the sensitivity of TPC test. TPC of the AWM powders was also significantly higher than the millet flour. The significantly higher (*p* < 0.05) TPC content of the AWM powders could be attributed to the additional phenolic compound that is present in AW and/or the interactions between whey protein and phenolic compounds^49,50^. In the case of DPPH activity, the AWM powders had similar DPPH activity with barnyard flour. The tannin content in Barnyard millet was 3.10 mg CAE/g and 2.34 mg CAE/g for Little millet. After spray drying, the tannin content in the AWM powders decreased to 2.12 mg CAE/g. This decrease could be attributed to tannins’ leaching during the feed solution’s processing and the synthesis of macromolecular compounds from phenolic compounds such as catechins. Similar tannin content was reported for whole finger and pearl millet varieties^51^. Tannins are polyphenols that form complexes with macromolecules, thereby affecting the bioavailability and utilization of these macromolecules and other nutrients. Moreover, tannins give the food an astringent taste and can reduce the absorption of vitamin B12, iron, and glucose^52^. These results indicated that the AWM powders retained antioxidant activity after spray drying with significantly reduced tannin content.

### Future applications of AWM powder

This study was conducted at a laboratory scale level, with plans to progress towards pilot-scale production of the optimized novel white powders. A pilot-scale study is crucial in assessing the potential of AWM powder as a food ingredient. This study would help determine the scalability of the production process, identify any potential challenges and maintain the nutritional and functional quality parameters of the AWM powder during scale up conditions. Conducting a pilot-scale study would ensure that AWM powder is a viable food ingredient that can meet market demands. In addition to the pilot-scale study, sensory analysis of AWM powder is necessary to evaluate consumer acceptability. The sensory analysis would involve testing the AWM powder for its taste, aroma, appearance, and texture, among other attributes. This information is essential for determining AWM powder’s marketability, identifying potential areas of improvement, and making necessary modifications to enhance its consumer appeal. The sensory analysis of the AWM powder would also depend on its intended food application. It is, therefore, essential to tailor sensory testing to the specific requirements of the target food or pharmaceutical product. This would ensure that the AWM powder meets the desired sensory characteristics for its intended food application and is acceptable to consumers.

**Figure 7:**
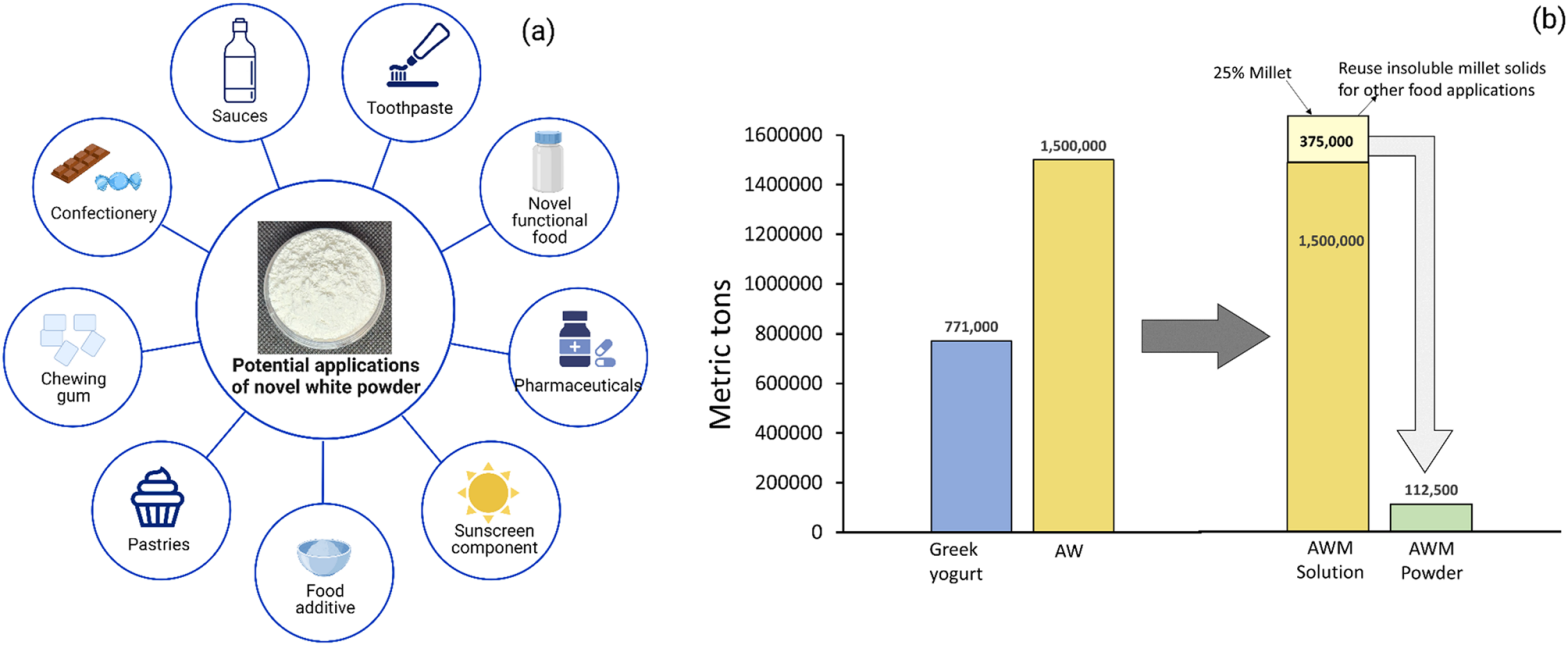
(**a**) Potential applications of novel millet acid whey powder. (**b**) Demonstrates the potential research impacts of producing AWM powder, based on assumptions derived from in-house laboratory experiments.

The novel AWM powder is rich in nutrients, minerals (such as P, K, Ca, Zn, and Cu), and antioxidants and could be extensively used in formulating functional foods and a mineral fortificant. Previous research^21^ has revealed that the physical properties of the AWM powder show it to be a highly soluble, free-flowing powder with good functional properties. These findings suggest that AWM powder has a wide range of potential food and non-food applications in the food and pharmaceutical industries (Fig. 7a). Some of those applications in the food sector could include being used in beverage formulation, sauces, or as a food coating to provide opacity and whiteness. In the pharmaceutical industry, AWM powder has potential applications as a vehicle for bioactive compounds, in developing clinical foods for people with special nutritional needs, and as a filler ingredient in pharmaceutical and food products^53^. With the growing concerns regarding the safety of the use of the food-grade whitener TiO_2_^54,55^ in food, there is an urgent need for food manufacturers and researchers to explore alternative food whitening agents. The white AWM powder is a promising ingredient that presents a natural, viable whitening alternative to TiO_2_ and could be utilized in a wide range of food and pharmaceutical products^53^. The usage or dosage of AWM powder in food applications would vary based on the intended application and target product. Different food products have different requirements regarding color, appearance, taste, functional properties, and nutritional content, which would affect the amount of AWM powder needed. For example, the amount of AWM powder needed for incorporating it into a beverage application would differ from that required for replacing TiO_2_ as a food coating material (e.g., in candies, sweets, chewing gums) or baked products (e.g., doughnuts, cakes, icing). Therefore, the dosage of AWM powder would need to be carefully calibrated to ensure that the target product meets its specific application requirements.

To fully understand the sustainability credentials of AWM powder, a life cycle assessment (LCA) would be necessary. An LCA is a comprehensive method for evaluating the environmental impacts of a product throughout its entire life cycle, from raw material acquisition to disposal^56^. It would provide a holistic view of the sustainability of AWM powder by considering factors such as resource use, energy consumption, and waste generation. LCA would help identify areas where improvements could be made to minimize the environmental impact of AWM powder production and usage, making it more sustainable in the long run. In addition to the LCA, a comparison study would be necessary to measure the economic feasibility of producing AWM powder. The study would compare the cost of current acid whey utilization (e.g., as animal feed or cost of treatment before disposal) to the cost of producing AWM powder for various food and non-food applications. This would enable researchers and manufacturers to determine the economic viability of producing AWM powder and whether it would be a cost-effective alternative to current acid whey utilization methods. Such a study would provide valuable information for determining the commercial potential of AWM powder and making decisions about its future development and production.

The utilization of AW in any of these applications could be promising as a valuable resource generator for the dairy industry. For instance, in 2015, ~ 1.5 million metric tons of AW were generated^5^. According to our in-house laboratory experiments, spray drying 100 ml of a 25% AWM solution yields ~ 7.5 g of powder. Extrapolating this to the annual production of 1.5 million metric tons of AW suggests that ~ 112,500 metric tons of 25% AWM powder could be produced (Fig. 7b). This has the potential to generate significant savings in transportation costs, reduce the environmental burden of the AW disposal process and create a new income stream for the dairy sector.

## Conclusion

As the global population continues to increase, it's essential to adopt sustainable approaches to food processing. Efforts to improve food systems in the dairy industry must incorporate sustainable agricultural practices and food processing operations that minimize food waste and food loss. Consumers have become more discerning about food ingredients and are increasingly drawn to natural labels, and AWM powder offers consumers a potential natural alternative to TiO_2_ in some food applications. However, the suitability of the AWM powder as a sustainable alternative to TiO_2_ would depend on the intended applications and future LCA. The AWM powder is highly soluble in water and could have some of the distinctive odor and flavor that is characteristic of AW, which must be considered when developing AWM based formulations. Therefore, sensory analysis with trained and consumer panels is essential in ensuring that AWM formulations meet consumers’ expectations.

The AWM powder developed in this study is an innovative white food powder made from underutilized grains and AW, offering the dairy sector a possible solution to the AW waste problem. Compositional analysis of the AWM powder revealed the retention of essential nutrients from millet and AW, indicating its potential for use in various food applications. The color analysis and nutritional information presented here could benefit scientists and the dairy sector in product formulation, while considering the nutritional value for consumers, the economic gain for the industry, and the environmental benefits for the public. Overall, this study provides a valuable contribution to the food industry by highlighting the potential of AWM powders as an opportunity to turn dairy waste into a valuable food ingredient while providing a safer and more sustainable alternative to TiO_2_ in some food applications.

## Data Availability

The datasets used and/or analysed during the current study are available from the corresponding author on reasonable request.
